# Redirecting E3 ubiquitin ligases for targeted protein degradation with heterologous recognition domains

**DOI:** 10.1016/j.jbc.2024.108077

**Published:** 2024-12-13

**Authors:** Huan Yang, Ge Zheng, Grace Y. Li, Alia Alshaye, Stuart H. Orkin

**Affiliations:** 1 Department of Pediatrics, Dana Farber/Boston Children’s Hospital Cancer and Blood Disorder Center, Boston, Massachusetts, USA; 2 Department of Pediatrics, Harvard Medical School, Boston, Massachusetts, USA; 3 Howard Hughes Medical Institute, Boston Children’s Hospital, Boston, Massachusetts, USA

**Keywords:** E3 ubiquitin ligases, targeted protein degradation, TRIM10, TRIM58, PROTACs, BCL11A

## Abstract

Targeted protein degradation (TPD) mediated by proteolysis targeting chimeras or molecular glues is an emerging therapeutic strategy. Despite greater than 600 E3 ligases and their associated components, a limited number have been deployed in TPD. Those commonly used include cereblon and von Hippel–Lindau tumor suppressor (VHL), which is expressed widely and for which high affinity ligands are available. Limiting TPD to specific cells or tissues would be desirable in many settings. To this goal we have determined the potential of two erythroid cell-enriched E3 ligases, TRIM10 and TRIM58, to degrade a protein of interest, BCL11A, a validated therapeutic target for the β-hemoglobinopathies. We established a general strategy in which heterologous recognition domains replace the PRY-SPRY domain of TRIM10 and TRIM58. Recruitment of TRIM10 or TRIM58 to BCL11A by coiled-coil peptides, nanobodies, or the substrate recognition domain of cereblon led to its degradation. Our findings illustrate a strategy that may be widely useful in evaluating the TPD potential of other E3 ubiquitin ligases and provide a rationale for discovery of ligands for TRIM10 and TRIM58 for erythroid-selective depletion of proteins of interest.

Targeted protein degradation (TPD) provides a means of reducing the level of a protein of interest (POI) for experimental or therapeutic purposes. Small molecules are employed to recruit ubiquitin ligases to the POI, acting either as molecular “glues” or heterobifunctional compounds (proteolysis targeting chimeras, PROTACs) (1). Induced proximity and complex formation between an E3 ubiquitin ligase and the POI promotes ubiquitylation of the target protein and degradation by the proteasome. PROTACs are comprised of a ligand binding to the POI and a second ligand that binds to and recruits an E3 ligase. The most thoroughly explored E3 ligases (or their components) for PROTACs are cereblon (CRBN) and von Hippel–Lindau tumor suppressor (VHL), for which high-affinity ligands are available (2). In the absence of a specific ligand for the POI, fusion proteins consisting of the POI and an artificial tag may be used, either expressed exogenously or from the endogenous locus. Of experimental platforms for TPD, the degradation tag (dTAG) system, which employs a ligand for a modified FKBP-binding protein in fusion with the POI, is versatile and has been used for target validation and assessment of immediate consequences of depletion of a POI (3, 4). CRBN and VHL-based PROTACs generally promote TPD in many different cells or tissues. While CRBN and VHL-based PORTACs have entered clinical trials (5), there are settings in which restricting TPD to a particular cell type or lineage would be beneficial and enhance safety by limiting effects in other cells.

With more than 650 known or predicted E3 ligases (and their associated components), their patterns of expression are diverse, and provide an opportunity for cell-restricted TPD by leveraging E3 ligases other than the few widely expressed ones that are in current use in PROTACs (6, 7, 8). Before embarking on a search for ligands for a given E3 ligase with a favorable gene expression profile, it would be valuable to demonstrate that it is capable of promoting TPD on recruitment to a POI. To this end, we have explored inducing proximity of a specific POI, BCL11A (9), with erythroid cell-enriched E3 ligases by replacing the E3 ligase recognition domain with different heterologous recruiting domains. We chose BCL11A as the POI because of its demonstrated role in fetal hemoglobin (HbF) silencing (10). TPD of BCL11A in erythroid precursors leads to reactivation of HbF expression (11). Moreover, erythroid-specific downregulation of BCL11A *via* CRISPR/Cas9 editing forms the basis of approved therapy for sickle cell disease and β-thalassemia (12, 13). Given the inherent challenges of treating large numbers of patients with editing therapies, as they are currently employed, small molecule drugs that promote TPD of BCL11A in erythroid cells would address a major unmet need. Restricting TPD to erythroid cells could mitigate potential effects of BCL11A loss in other cells or tissues. Here, we surveyed E3 ligases and selected those expressed in erythroid cells and then tested several recruitment strategies. Our findings demonstrate that recruitment of either TRIM10 or TRIM58 to BCL11A induces TPD.

## Results

### TRIM10 and TRIM58 as erythroid-enriched E3 ubiquitin ligases

**Figure 1:**
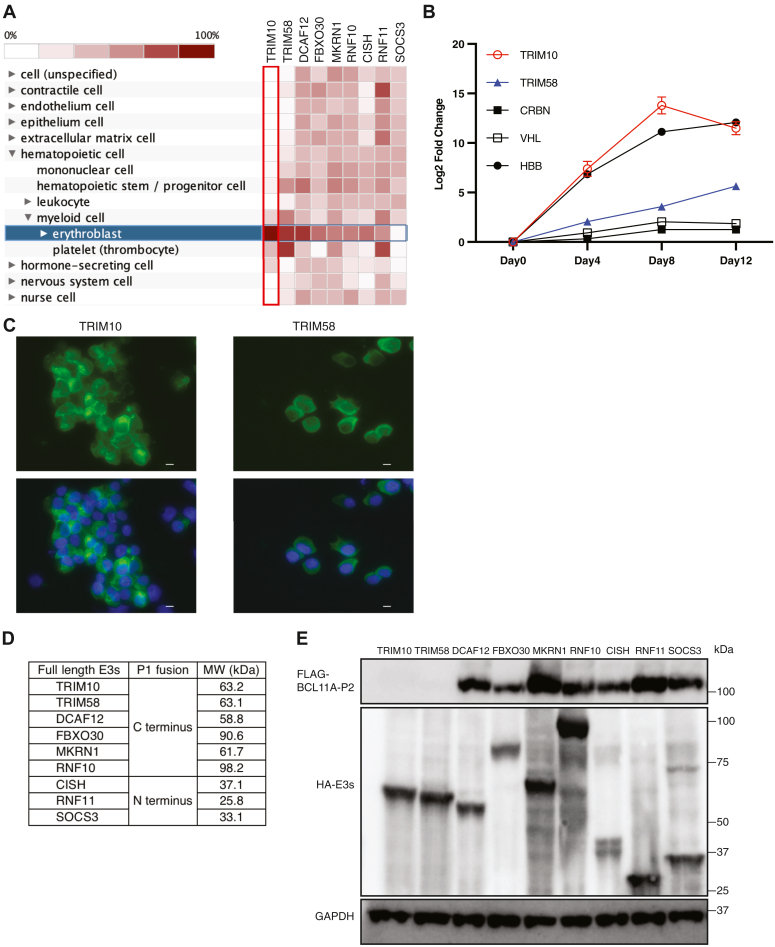
**TRIM10 and TRIM58, modular TRIM proteins, are selectively expressed in the erythroid lineage.***A*, heat map representing relative RNA expression of E3 ligases (or components) that are upregulated during erythroid differentiation. Genevestigator dataset HS_mRNASeq_HUMAN_GL-0 was analyzed. Colors from *white* to *burgundy* indicate from the minimum to the maximum expression in the dataset. *B*, RNA expression kinetics of TRIM10, TRIM58, CRBN, VHL, and HBB during *in vitro* erythroid differentiation of CD34^+^ cells. The values represent means ± SD, n = 3 independent repeats. *C*, subcellular localization of TRIM10 and TRIM58. 3xHA-tagged full-length TRIM10 and TRIM58 were exogenously expressed in HUDEP2 cells. Anti-HA primary antibody and FITC-conjugated secondary antibody were used for Immunofluorescence. Nuclei were stained with 4′,6-diamidino-2-phenylindole. The scale bar represents 10 μm. *D*, full-length E3 ligase-P1 fusion proteins tagged at C or N terminus. *E*, BCL11A-P2 levels in the presence of full-length E3 ligase-P1 fusion proteins. Constructs of BCL11A-P2 and full length E3 ligase-P1 were cotransfected into HEK293T cells. 24 h later, cells were lysed for Western blot. Molecular weight markers are indicated. Full-length E3 ligase-P1 proteins were tagged with 3xHA to monitor their expression. CRBN, cereblon; HBB, hemoglobin beta; VHL, von Hippel–Lindau tumor suppressor.

We first examined the gene expression pattern of E3 ligases, including those that have been reported to be upregulated during erythroid cell maturation (14) (Figs. 1*A* and S1). The TRIM family members TRIM10 and TRIM58 were the most selectively expressed in erythroid cells. Both are induced in expression during *in vitro* erythroid cell maturation of primary human CD34^+^ cells (Fig. 1*B*). As assessed by expression of HA-tagged constructs in HUDEP2 cells (15), a human umbilical cord-derived erythroid progenitor cell line, TRIM10 appeared to localize to both cell membrane and cytoplasm, while TRIM58 was predominantly cytoplasmic (Fig. 1*C*). TRIM10, initially described as HERF1, was proposed as a factor required for erythroid differentiation (16). However, Trim10^−/−^ mice appear to be viable (17). TRIM58 promotes degradation of dynein during terminal maturation (18) and has been associated with red cell size in genome-wide association studies (19).

In initial experiments, we used coiled-coil peptides to recruit full-length TRIM10 and TRIM58, as well as the other E3 proteins that are upregulated during erythroid differentiation, to BCL11A. The P1 and P2 peptides form heterodimers with high affinity *via* the interaction of polypeptide helices along their complementary surfaces (20). The small size (33 amino acids) of the coiled-coil peptides minimizes potential adverse effects of their attachment to tagged proteins. Based on the organization of each candidate protein, we generated constructs expressing proteins with P1 fused to either the N or C terminus (Fig. 1*D*). Constructs were cotransfected with C terminally P2-tagged BCL11A complementary DNA into HEK293T cells. As shown by Western Blot, the level of BCL11A-P2 was reduced strikingly only by TRIM10 and TRIM58 P1 fusion proteins (Fig. 1*E*). This observation suggested that among this set of erythroid-induced factors, TRIM10 and TRIM58, are most readily repurposed for TPD, perhaps due to the presence of a long, flexible coiled-coil domain between the E2 ubiquitin conjugating enzyme binding (N-terminus) and substrate recognition (C-terminus) domains.

### General strategy for redirecting TRIM proteins to a POI

**Figure 2:**
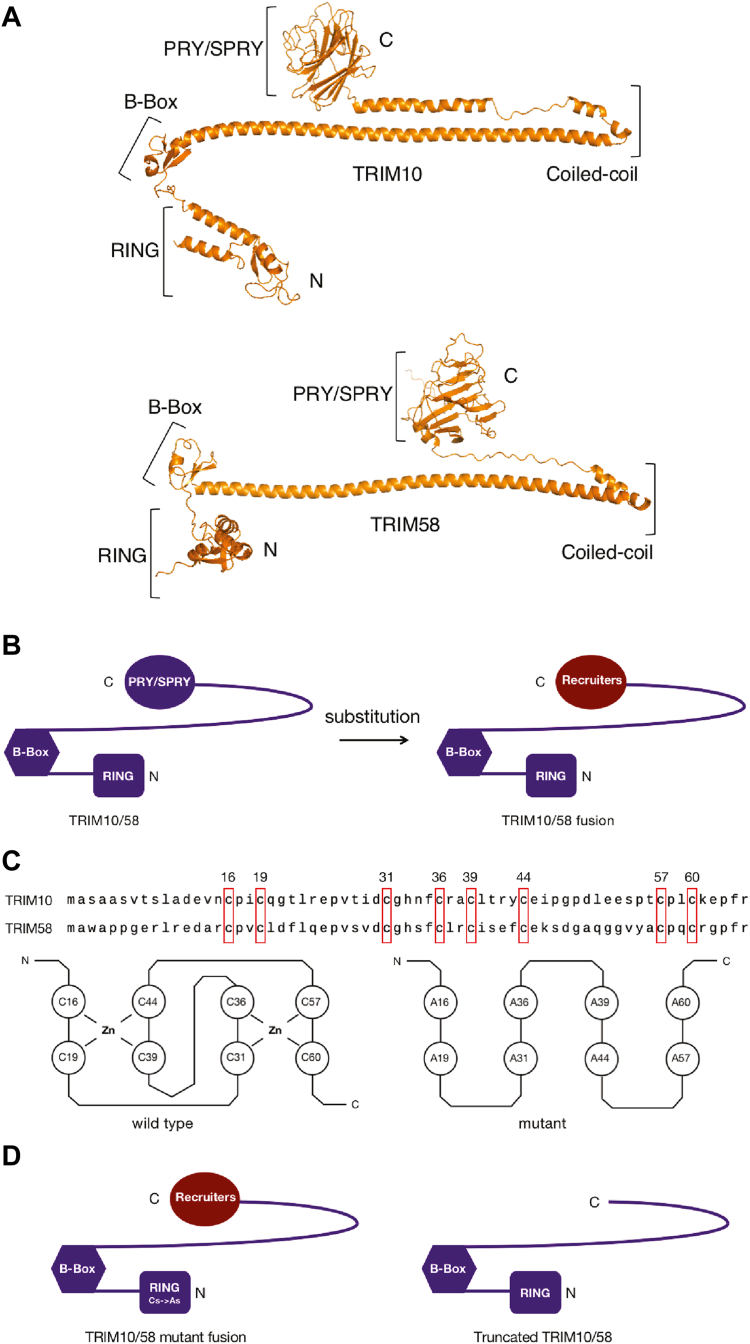
**Substitution and mutational strategies based on the modular structures of TRIM10 and TRIM58.***A*, domain structures of TRIM10 and TRIM58 predicted by AlphaFold2 (38). Structures were retrieved from AlphaFold Protein Structure Database. TRIM10, A0A2K6ARI5 (A0A2K6ARI5_MACNE). TRIM58, A0A2K6F3G9 (A0A2K6F3G9_PROCO). *B*, general strategy of replacing the TRIM PRY-SPRY domain with a heterologous-recruiting domain. *C*, the sequences of conserved RING domain and mutations introduced to inactive TRIM proteins. The eight conserved cysteine residues which form the C3HC4 zinc finger are highlighted with *red boxes*. The eight conserved cysteine residues were mutated to alanine residues to disrupt the zinc-finger structure. *D*, schematics of the two controls: TRIM mutant fusion protein and TRIM truncated protein without recruiters.

TRIM10 and TRIM58 proteins are modular, as depicted in Figure 2*A*. Each consists of C3HC4 zinc finger RING, B-Box, coiled-coil, and PRY/SPRY domains (21, 22). The PRY-SPRY domain confers substrate recognition (23, 24). Therefore, we explored replacement of this domain with heterologous domains to bring the POI, BCL11A, in proximity (Fig. 2*B*). The use of different types of recognition domains provides a level of assurance that the results obtained reflect the potential of the TRIM proteins to degrade the target protein. Moreover, the approach we have used should be applicable to testing other E3 ubiquitin ligases prior to embarking on a ligand discovery campaign. We generated constructs in which the eight conserved cysteines in the RING domain were mutated to alanine residues to disrupt binding of TRIM10 and TRIM58 to E2 ubiquitin-conjugating enzymes and render them catalytically inactive (25) (Fig. 2*C*). Mutant fusion constructs and truncated TRIM constructs were used as controls in subsequent experiments (Fig. 2*D*).

### Recruitment of TRIM10 and TRIM58 with coiled-coil peptides

**Figure 3:**
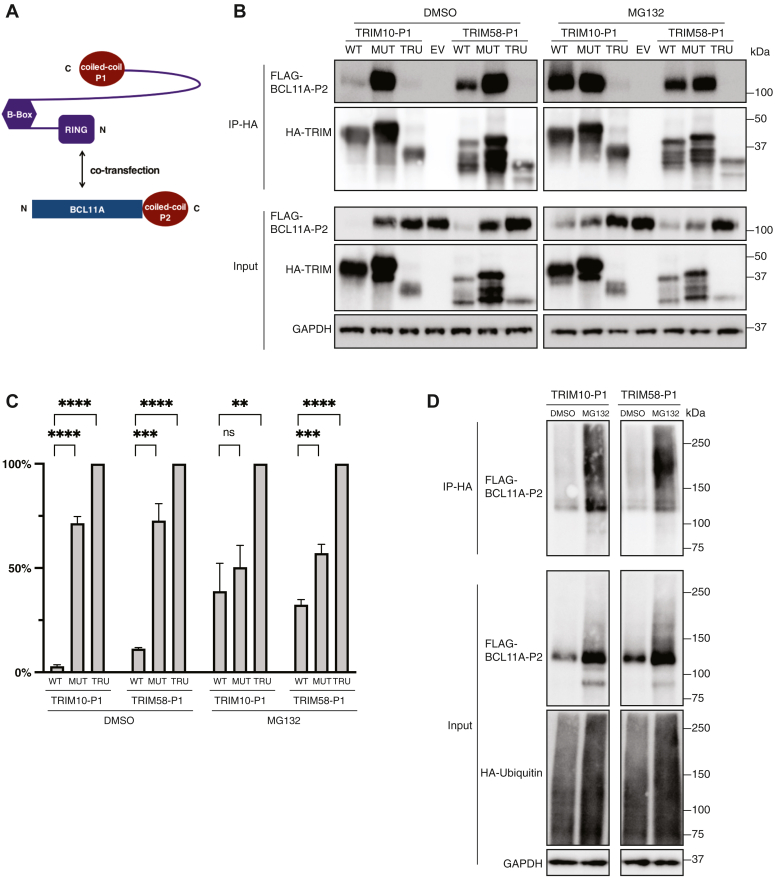
**Replacement of the PRY-SPRY domain of TRIM10 and TRIM58 with coiled-coil peptide allows recruitment to and degradation of BCL11A.***A*, schematic for cotransfection of TRIM10/58-P1 and BCL11A-P2. *B*, Western Blot of cotransfection, MG132 treatment and coimmunoprecipitation. Twenty-four hours post cotransfection, 10 μM MG132 or equivalent volume of DMSO was added. Sixteen hours later, cells were lysed for coimmunoprecipitation. TRIM10/58-P1 proteins were tagged with 3xHA. BCL11A-P2 protein was tagged with 3xFLAG. WT fusion proteins; MUT, mutant fusion proteins; TRU, truncated TRIM proteins; EV, empty vector–expressing mCherry. *C*, quantitation of Western blot in B (input of BCL11A-P2). The protein level of BCL11A-P2 in cotransfection with the truncated protein construct was set as 100%. *D*, ubiquitylation assay with coexpression of HA-ubiquitin, 3xFLAG-BCL11A-P2, and 6xHis-TRIM-P1 constructs. The values in statistical graphs represent means ± SD, n = 3 independent repeats, unpaired *t* test was used for the calculation of *p* values, ∗∗∗∗*p* < 0.0001; ∗∗∗*p* < 0.001; and ∗∗*p* < 0.01, ns not significant.

As the next step in evaluating the functional potential of TRIM10 and TRIM58, we replaced their respective PRY/SPRY domains with P1 (Fig. 3*A*). Upon cotransfection into HEK293T cells, compared to the mutant fusion proteins and truncated proteins, TRIM-P1 fusion proteins led to a significantly reduced level of BCL11A-P2 (Fig. 3, *B* and *C*, input dimethyl sulfoxide [DMSO] section). We found that the mutant fusion proteins retained some activity, indicating the eight cysteine-to-alanine mutations may not have abolished the binding between TRIM proteins and E2 ubiquitin-conjugating enzymes. Treatment with the proteasome inhibitor MG132 (26) partially restored the level of BCL11A-P2 protein (Fig. 3, *B* and *C*, input MG132 section). We detected direct interaction between the TRIM-P1 fusion proteins and BCL11A-P2 (Fig. 3*B*, immunoprecipitation-HA DMSO section). In MG132-treated cells, TRIM-P1 fusion proteins pulled down much more BCL11A-P2 than in DMSO-treated cells (Fig. 3*B*, immunoprecipitation-HA MG132 section). We performed an assay for ubiquitylation of BCL11A-P2 by coexpression of ubiquitin and TRIM-P1 constructs. Upon the MG132 treatment, we observed a size shift of BCL11A-P2 and accumulation of ubiquitinated BCL11A-P2 (Fig. 3*D*). Taken together, our findings indicate that TRIM-P1 fusion proteins promote TPD *via* the proteosome.

### Recruitment of TRIM10 and TRIM58 with nanobodies and PROTACs

**Figure 4:**
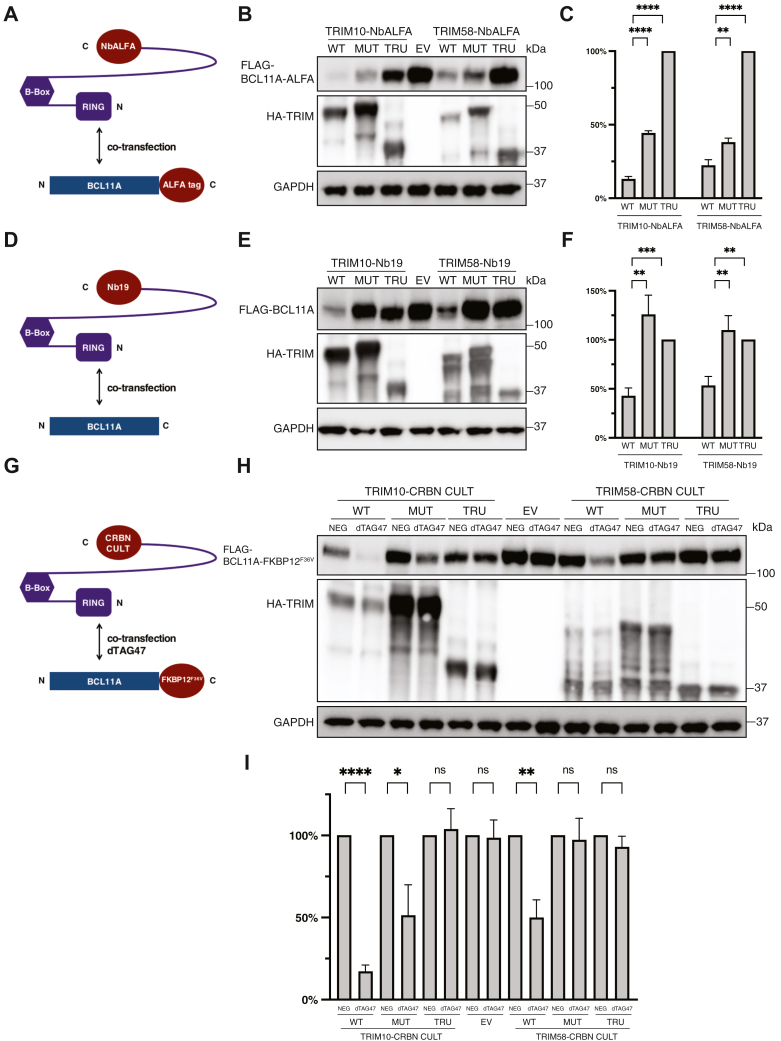
**Nanobodies and the CRBN-CULT domain recruit TRIM10 and TRIM58 to BCL11A for TPD.***A*, cotransfection of TRIM10/58-NbALFA and BCL11A-ALFA into HEK293T cells. *B*, Western blot of lysates prepared at 24 hours after cotransfection, as illustrated in *A*. TRIM10/58-NbALFA proteins were tagged with 3xHA. *C*, quantitation of Western blot in *B*. The protein level of BCL11A-ALFA in cotransfection with the mutant construct was set as 1. *D*, cotransfection of TRIM10/58-Nb19 and BCL11A into HEK293T cells. *E*, Western blot of lysates prepared at 24 h after cotransfection, as illustrated in *D*. TRIM10/58-Nb19 proteins were tagged with 3xHA. *F*, quantitation of Western blot in *D*. The protein level of BCL11A in cotransfection with the mutant construct was set as 1. *G*, cotransfection of TRIM10/58-CULT and BCL11A-FKBP12^F36V^ into HEK293T cells. 0.5 μM dTAG-47 and an inactive analog (dTAG-47-NEG) were added at the time of cotransfection. HEK293T cells were lysed for Western blot 24 h post transfection. *H*, Western blot of lysates prepared at 24 h after cotransfection, as illustrated in *G*. TRIM10/58-CULT proteins were tagged with 3xHA. *I*, quantitation of Western blot in *H*. The protein level of BCL11A-FKBP12^F36V^ in cotransfection with mutant construct was set as 1. The values in the statistical graphs represent means ± SD, n = 3 independent repeats, unpaired *t* test was used for the calculation of *p* values, ∗∗∗∗*p* < 0.0001; ∗∗∗*p* < 0.001; ∗∗*p* < 0.01; and ∗*p* < 0.05, ns not significant. CRBN, cereblon; TPD, targeted protein degradation.

While these experiments indicated that TRIM10 and TRIM58 are capable of eliciting degradation of BCL11A, the stability of interactions mediated by coiled-coil peptides may not mimic those of conventional PROTACs. As a second test, we replaced the recognition domain of TRIM10 and TRIM58 with two different nanobodies. In the first instance, we used a high-affinity (∼26 pM) nanobody to the ALFA tag (27) (Fig. 4*A*). Coexpression of TRIM-NbALFA fusion proteins with BCL11A tagged at the C terminus with ALFA resulted in degradation of BCL11A protein, as assessed by Western Blot (Fig. 4, *B* and *C*). Similarly, nanobody Nb19 directed to the DNA-binding domain of BCL11A (28) with an affinity of ∼20 nM directed degradation of untagged BCL11A protein upon cotransfection (Fig. 4, *D*–*F*). The differing extents of reduction of BCL11A protein may reflect the relative affinities of NbALFA and Nb19.

To more closely approximate a PROTAC, we next substituted the PRY-SPRY domains of TRIM10 and TRIM58 with the substrate recognition domain (CULT, CRBN domain of unknown activity, binding cellular ligands, and thalidomide) of CRBN (Fig. 4*G*). Transfer of this domain to the TRIM proteins mimics the dTAG platform in which bivalent PROTACs recruit CRBN to a POI expressed in fusion with FKBP12^F36V^. Previously we used the dTAG platform for TPD of BCL11A in erythroid cells to examine the immediate consequences of BCL11A loss on target gene transcription (11). In order to avoid confounding endogenous CRBN, we generated CRBN KO HEK293T cells by CRISPR/Cas9 editing (Fig. S2). TRIM-CRBN fusions (either WT TRIM or mutated TRIM) and BCL11A-FKBP12^F36V^ were coexpressed in CRBN KO HEK293T cells. As shown in Figure 4, *H* and *I*, addition of dTAG-47 led to degradation of tagged BCL11A, greater with the TRIM10 fusion than TRIM58. We determined the degradation kinetics for TRIM10-CRBN upon the treatment of dTAG-47. Maximal degradation was achieved by about 8 h (Fig. S3). As an additional control, we employed an inactive version of dTAG-47 (NEG) (29), which failed to reduce the BCL11A level (Fig. 4, *H* and *I*). Taken together, these experiments demonstrate that the recognition domain of CRBN can be repurposed to direct recruitment of an E3 ligase, such as TRIM10 and TRIM58, to a POI for TPD with a PROTAC.

### Degradation of BCL11A-ALFA by TRIM10-NbALFA at endogenous level

**Figure 5:**
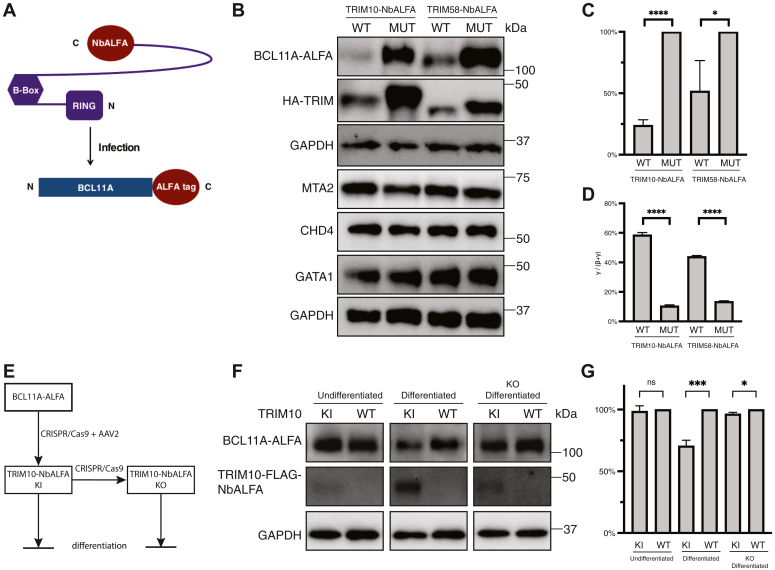
**TRIM10/58-NbALFA fusion proteins degraded endogenous BCL11A-ALFA proteins, leading to reactivation of γ-globin expression.***A*, infection of biallelic BCL11A-ALFA knock-in HUDEP2 cells with lentiviral TRIM10/58-NbALFA constructs. Transduction was monitored by IRES-mCherry. After infection, mCherry^+^ cells were purified by FACS, and then cultured in erythroid differentiation medium for 7 days before analysis. *B*, Western blot of experiment in A. TRIM10/58-NbALFA proteins were tagged with 3xHA. BCL11A-ALFA was blotted with BCL11A antibody. MTA2, CHD4 and GATA1 were blotted with their antibodies, respectively. *C*, quantitation of BCL11A-ALFA levels in *B*. The protein level of BCL11A-ALFA in the truncated protein construct infected cells was set as 100%. *D*, percent of γ-globin *versus* γ + β globin RNA transcripts. *E*, schematic depicting the experiments with endogenously expressed TRIM10-NbAFLA. The TRIM10-NbAFLA knockin is monoallelic. *F*, Western blot of experiments illustrated in *E*. *G*, quantitation of Western blot in *F*. The values in the statistical graphs represent means ± SD, n = 3 independent repeats, unpaired *t* test was used for the calculation of *p* values, ∗∗∗∗*p* < 0.0001; ∗∗∗*p* < 0.001; and ∗*p* < 0.05; ns not significant. FACS, fluorescence-activated cell sorting.

Finally, we asked if TRIM10 and TRIM58 are capable of degrading endogenously expressed BCL11A in erythroid cells, and as a consequence reactivating expression of HbF. To this end, we used HUDEP2 cells (15). These cells model adult-type erythroid differentiation in culture (low γ-globin and high β-globin expression) (30). We generated HUDEP2 cells in which the ALFA tag was targeted to the C terminus of both BCL11A alleles by CRISPR/Cas9 editing (Fig. S4), thereby ensuring a normal level of expression. Cells infected with lentivirus-expressing TRIM10- or TRIM58-NbALFA, or their TRIM-mutant counterparts, were purified and differentiated for 7 days (Fig. 5*A*). As shown in Figure 5, *B* and *C*, WT TRIM10- and TRIM58-NbALFA fusions, but not their respective mutant counterparts, markedly reduced the level of BCL11A protein on day 7 of differentiation, leading to induction of γ-globin transcripts (Fig. 5*D*). The reduction of BCL11A protein was specific, as the levels of transcription factor GATA1 and components of the nucleosome remodeling and deacetylase complex (MTA2, CHD4), which functions with BCL11A in repression, were unaffected (Fig. 5*B*).

Since overexpression of TRIM10-NbALFA led to a stronger decrease in BCL11A level than TRIM58-NbALFA, we generated a TRIM10-NbALFA knock-in allele in BCL11A-ALFA HUDEP2 cells (Fig. S5*A*). We used these cells to ask if TRIM10-NbALFA can degrade BCL11A-ALFA (Fig. 5*E*). Note that in these cells, both fusion proteins are expressed from the endogenous loci. BCL11A-ALFA level was comparable in undifferentiated TRIM10-NbALFA knock-in cells compared to WT cells due to low expression level of TRIM10-NbALFA. After the induction of differentiation, TRIM10-NbALFA was significantly upregulated at RNA (Fig. S5*B*) and protein levels (Fig. 5*F*), and the level of BCL11A-ALFA was modestly reduced. To test if this finding was due to expression of TRIM10-NbALFA, we performed CRSIPR/Cas9 editing to KO TRIM10. The TRIM10-NbALFA knockout restored the BCL11A-ALFA level (Fig. 5, *F* and *G*). Thus, when brought into proximity, endogenously expressed TRIM10-NbALFA is able to promote degradation of endogenously expressed BCL11A-ALFA in erythroid cells.

## Discussion

Restricting TPD to specific cell types would expand the therapeutic options for PROTACs by limiting undesired effects of widespread protein depletion. Here, we have used a general strategy to assess the capability of two erythroid cell–enriched E3 ubiquitin ligases, TRIM10 and TRIM58, to degrade a POI, BCL11A, which is a validated target for reactivation of HbF in hemoglobin disorders. Given roles for BCL11A in neurodevelopment, B-lymphocyte differentiation, and maintenance of hematopoietic stem cells (31, 32, 33), approaches to mitigating consequences of functional impairment of BCL11A in nonerythroid cells are of interest. To test TPD mediated by TRIM10 and TRIM58, we have replaced their PRY-SPRY substrate recognition domains with coiled-coil peptides, nanobodies, and the recognition domain of CRBN to induce physical proximity with BCL11A. In each instance, we observed that these E3 ligases are capable of promoting TPD of BCL11A and would likely do so with a variety of protein targets. Indeed, TRIM58 has been reported to degrade several targets in cells, including the dynein complex in late erythroid cells (18, 34, 35). The use of different recruiting elements with varying affinities suggests that the domain structure of TRIM10 and TRIM58 may be particularly favorable for TPD of novel target proteins. Our strategy of replacing the substrate recognition domain to redirect TRIM10 and TRIM58 to a POI is likely to be useful in evaluating other cell-enriched E3 ligases. To our knowledge, we are unaware of other reports in which the substrate recognition domain of CRBN has been used to confer dTAG-mediated TPD of a target protein.

While the demonstration that TRIM10 and TRIM58-mediated TPD of BCL11A elicits reactivation of γ-globin expression in erythroid cells when expressed as fusions with a nanobody is encouraging, many issues remain to be addressed in future studies. By necessity, our experiments have relied on exogenous expression of TRIM-fusion constructs. Are either of these E3 ligases, as expressed normally in cells, sufficiently abundant or accessible to BCL11A to promote TPD? What affinities of a bifunctional PROTAC for the TRIM and target protein are required for efficient TPD? These questions are best addressed when ligands for the TRIMs and specific target proteins become available. Our findings support further consideration of TRIM10 and TRIM58 as candidates for erythroid-selective TPD. To this end, efforts to develop small molecule ligands for these E3 ligases (36) should be encouraged.

## Experimental procedures

### Identification of erythroid cell–enriched E3 ligases

Expression patterns in cell types of E3 ligases and their associated components were analyzed with GENEVESTIGATOR, platform: mRNA-Seq Gene Level *Homo sapiens* (ref:Ensemble 97, GRCh38.p12).

### Erythroid differentiation of CD34^+^ cells

CD34^+^ cells were thawed, seeded in CD34 expansion medium at 100,000 cells/ml and cultured for 4 days. The cells were spun down, washed once with PBS, seeded in CD34 differentiation medium 1 at 100,000 cells/ml, and cultured for 4 days. Fresh CD34 differentiation medium 1 was added on day 4 to adjust the cell density to 100,000 cells/ml. Cells were transferred to CD34 differentiation medium 2 on day 8. Cells were collected on day 12 for analysis. Details of the culture media can be found in Table S1.

### Reverse transcription and quantitative PCR

RNA was extracted with RNeasy Plus Mini Kit (Qiagen, 74134). Reverse transcription was performed with QuantiTect Reverse Transcription Kit (Qiagen, 205311). Primer information can be found in Table S2. Quantitative PCR was performed in 384-well plate with Bio-Rad CFX384 real-time system under condition: 95 °C for 30 s, 95 °C for 10 s, 55 °C for 30 s, 72 °C for 30 s, 40 cycles, and 72 °C for 5 min.

### Immunofluorescence

100,000 cells were washed with PBS twice and resuspended in 100 ul 1% BSA PBS. After cytospin, monolayer cells were fixed with methanol for 5 min, air dry, incubated with 4% milk TBST (1X tris-buffered saline with 0.1% Tween) for 30 min for blocking and incubated with anti-HA antibody (Abcam, ab236632) overnight at 4 °C. The cells were washed with TBST three times for 5 min each, and incubated with FITC-conjugated secondary antibody (Thermo Fisher Scientific, 65-6111) for 1 h at room temperature (RT). The cells were washed with TBST three times for 5 min each. Nuclei were stained with 4′,6-diamidino-2-phenylindole. The slides were mounted for microscopic observation.

### Plasmid construction and cotransfection

The coding cassettes of TRIM fusion proteins and BCL11A fusion proteins were cloned into lentiviral vector pLVX under EF1a promoter and EFS promoters, followed by IRES-mCherry and IRES-eGFP, respectively. In the degradation experiments, the TRIM fusion proteins were tagged with 3xHA and BCL11A fusion proteins were tagged with 3xFLAG at the N terminus to detect their protein level in immunoblotting (Table S3). To transfect HEK293T cells in one well of 12-well plate, 0.5 μg of TRIM construct and 0.5 μg of BCL11A construct were mixed in 44 ul Dulbecco's modified Eagle's medium (DMEM). 5.5 μg polyethylenimine was added. Samples were vortexed and incubated at RT for 30 min. Then, the reaction was added to HEK293T cells. In the ubiquitylation assay, ubiquitin, BCL11A-P2 and TRIM-P1 were tagged with HA, 3xFLAG, and 6xHis, respectively. To transfect HEK293T cells in one well of 6-well plate, 1 μg of ubiquitin construct, 1 ug of TRIM construct, and 1 μg of BCL11A construct were mixed in 120 ul DMEM, followed by addition of 15 μg polyethylenimine. Twenty-four hours post cotransfection, 10 μM MG132 or equivalent volume of DMSO was added. Sixteen hours later, cells were lysed.

### Coimmunoprecipitation

Anti-HA (Thermo Fisher Scientific, 26181) or Anti-FLAG (GenScript, L00432) agarose resins were washed three times with tris-buffered saline. Cells were lysed with radio-immunoprecipitation assay buffer supplemented with protease inhibitor cocktail. One-tenth of the lysate was aliquoted as input. The rest of the lysate was incubated with the resins for 2 h at RT. The resins were washed three times with TBST and resuspended in 1× loading buffer. The proteins were released from the resins by boiling 5 min.

### Immunoblotting

Cells were washed twice with PBS and lysed with radio-immunoprecipitation assay buffer (Boston BioProducts, BP-115) supplemented with 1× protease inhibitor cocktail (Thermo Fisher Scientific, 87786). Proteins were separated with the 4 to 20% Criterion TGX Precast Midi Protein Gel (Bio-Rad, 5671095) according to the manufacturer’s protocol. Trans-Blot Turbo Transfer System was used to transfer the proteins to a polyvinylidene fluoride (PVDF) membrane. The PVDF membrane was blocked in 4% milk TBST for 30 min, washed three times with TBST, 5 min each time, and incubated with primary antibody at the recommended concentration overnight at 4 °C. The PVDF membrane was washed three times with TBST, 5 min each, and incubated with horseradish peroxidase-conjugated secondary antibody in 4% milk 1× TBST for 1 h, RT. The PVDF membrane was washed three times with TBST, 5 min each. Proteins were detected with chemiluminescence according to the manufacturer’s protocol (PerkinElmer, NEL104001EA). Details of the antibodies can be found in Table S4. Blots were quantitatively analyzed with ImageJ (https://imagej.net/ij/). The signals were normalized according to GAPDH level.

### HUDEP2 cell culture and differentiation

HUDEP2 cells were cultured in HUDEP2 expansion medium. To induce differentiation, cells were washed with PBS and resuspended in the HUDEP2 differentiation medium at 50, 000 cells/ml. Fresh differentiation medium was replenished on day 4. Cells were collected on day 7 for analysis. Details of the media can be found in Table S1.

### Virus preparation and infection

Viruses were prepared in 6-well plates. Two micrograms lentiviral vector, 1.5 μg psPAX2, and 0.5 μg pCMV-VSV-G were mixed in 160 μl DMEM. Twenty micrograms polyethylenimine was added into the reaction followed by vortexing. Reactions were incubated for 30 min at RT and added to HEK293T cells. The medium was changed the next day. Viruses were collected on day 1 and day 2 after the medium change. HUDEP2 cells were spinfected at 500*g*, 2 h, 30 °C. The medium was changed after the spinfection. Two days later, mCherry+ cells were purified by fluorescence-activated cell sorting.

### Ribonucleoprotein electroporation to HEK293T cells and HUDPE2 cells

50, 000 cells were collected by centrifugation at 500*g* for 5 min, washed once with PBS, and resuspended in 20 μl mixture of Nucleofector Solution and Supplement. Cas9/single-guide RNA ribonucleoprotein was generated by mixing 120 pM modified guide RNA (Synthego) (Table S5) with 61 pM Cas9-Alt-R protein (IDT) and 1 μl of Alt-R Cas9 Electroporation Enhancer (IDT), and incubated at RT for 10 min. Ribonucleoprotein was then mixed with cells, and electroporation was carried out in a 4D-nucleofector X Unit (Lonza). In knock-in experiment, after electroporation, 20 ul of crude rAAV viral prep was added to the HUDEP2 cells (11). After 4 to 6 days of culture, ∼40 cells were seeded per 96-well plate to isolate single cell clones. PCR was performed followed by Sanger sequencing to screen for clones with correct genetic modifications (Table S6).

## Data availability

Data supporting this article are included within the main text. All information will be shared upon request.

## Supporting information

This article contains supporting information (37).

## Conflict of interest

S. H. O. is an Investigator of the Howard Hughes Medical Institute. The other authors declare that they had no conflicts of interest with the contents of this article.
