# Supplementary material for: Redirecting E3 ubiquitin ligases for targeted protein degradation with heterologous recognition domains
Source: J Biol Chem. 2024 Dec 13;301(1):108077. doi: 10.1016/j.jbc.2024.108077 (PMC11758572; doi:10.1016/j.jbc.2024.108077)
Supplement: Supporting information [file mmc1.docx]

**Supporting information**

**Redirecting E3 Ubiquitin Ligases for Targeted Protein Degradation with Heterologous Recognition Domains**

**Running title: Redirecting E3 Ubiquitin Ligases**

**Keywords: E3 Ubiquitin Ligases, targeted protein degradation, TRIM10, TRIM58, PROTACs, BCL11A**

**Huan Yang^1,2^, Ge Zheng^1,2^, Grace Y. Li^1^, Alia Alshaye^1^, Stuart H. Orkin^1,2,3,*^**

^1^Dana Farber/Boston Children’s Hospital Cancer and Blood Disorder Center, Boston, MA, 02115; ^2^Harvard Medical School, Boston, MA, 02115; ^3^Howard Hughes Medical Institute, Boston Children’s Hospital, Boston, MA 02115.

*For correspondence: Stuart H. Orkin, [stuart_orkin@dfci.harvard.edu](mailto:stuart_orkin@dfci.harvard.edu)

Supplementary Figure S1


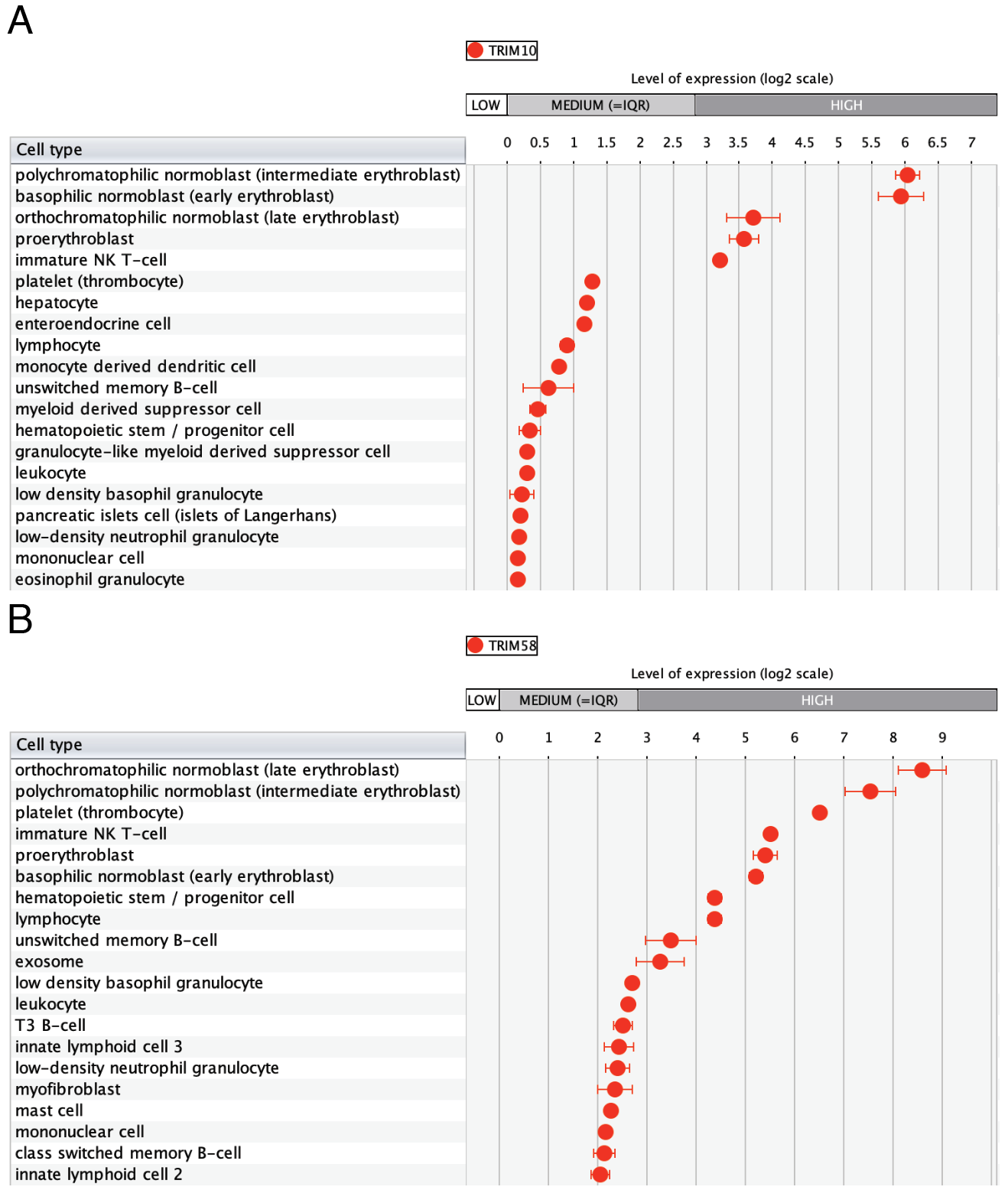


Figure S1. Expression pattern of TRIM10 (A) and TRIM58 (B). Genevestigator data set HS_mRNASeq_HUMAN_GL-0 was used. Top 20 cell types ranked by expression level of TRIM10 or TRIM58 are shown.

Supplementary Figure S2


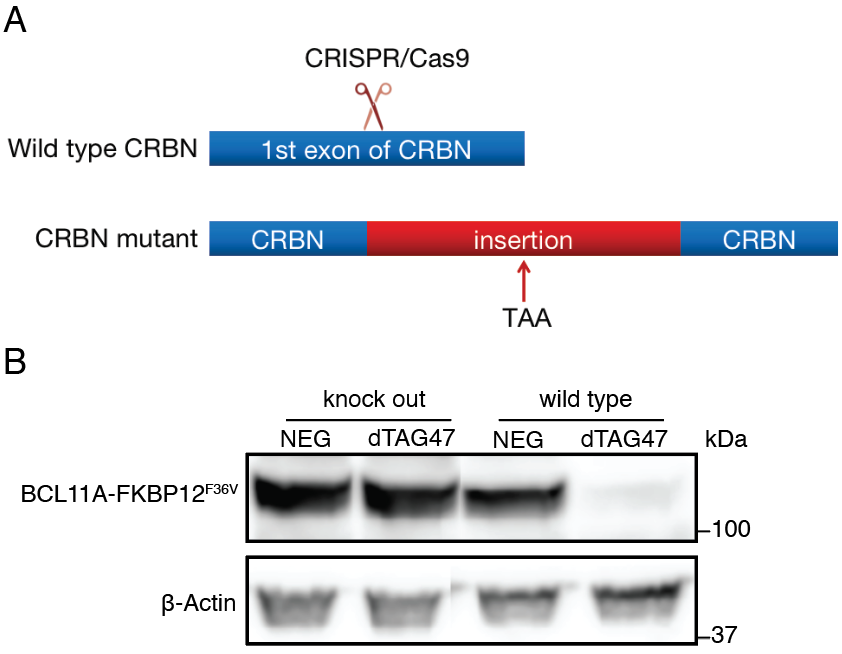


Figure S2. Generation of CRBN knock-out HEK293T cells. A, CRBN knock-out strategy. In the CRBN knock-out HEK293T cell line, an insertion introduces an early stop codon, resulting in the production of a short peptide. B, validation of the CRBN knock out HEK293T cell line. The BCL11A-FKBP12^F36V^ construct was transfected into the knock-out and wild type cells. dTAG47 or an inactive counterpart (NEG) was added at 0.5 µM. Cells were collected 24 hours later for Western Blot.

Supplementary Figure S3


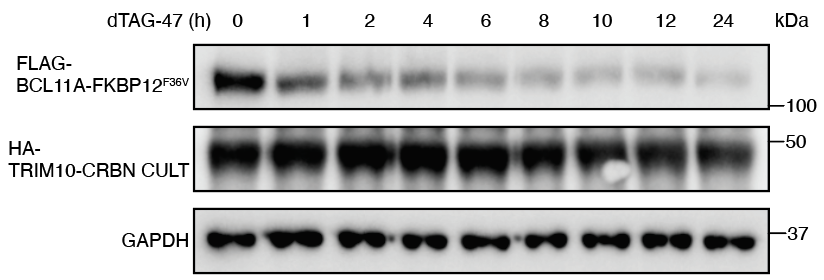


Figure S3. Degradation kinetics for TRIM10-CRBN. 24 hours post co-transfection of TRIM10-CRBN and BCL11A-FKBP12^F36V^, 0.5uM dTAG-47 was added. Cells were collected at the indicated time points. BCL11A-FKBP12^F36V^ and TRIM10-CRBN were blotted with FLAG and HA antibodies, respectively.

Supplementary Figure S4


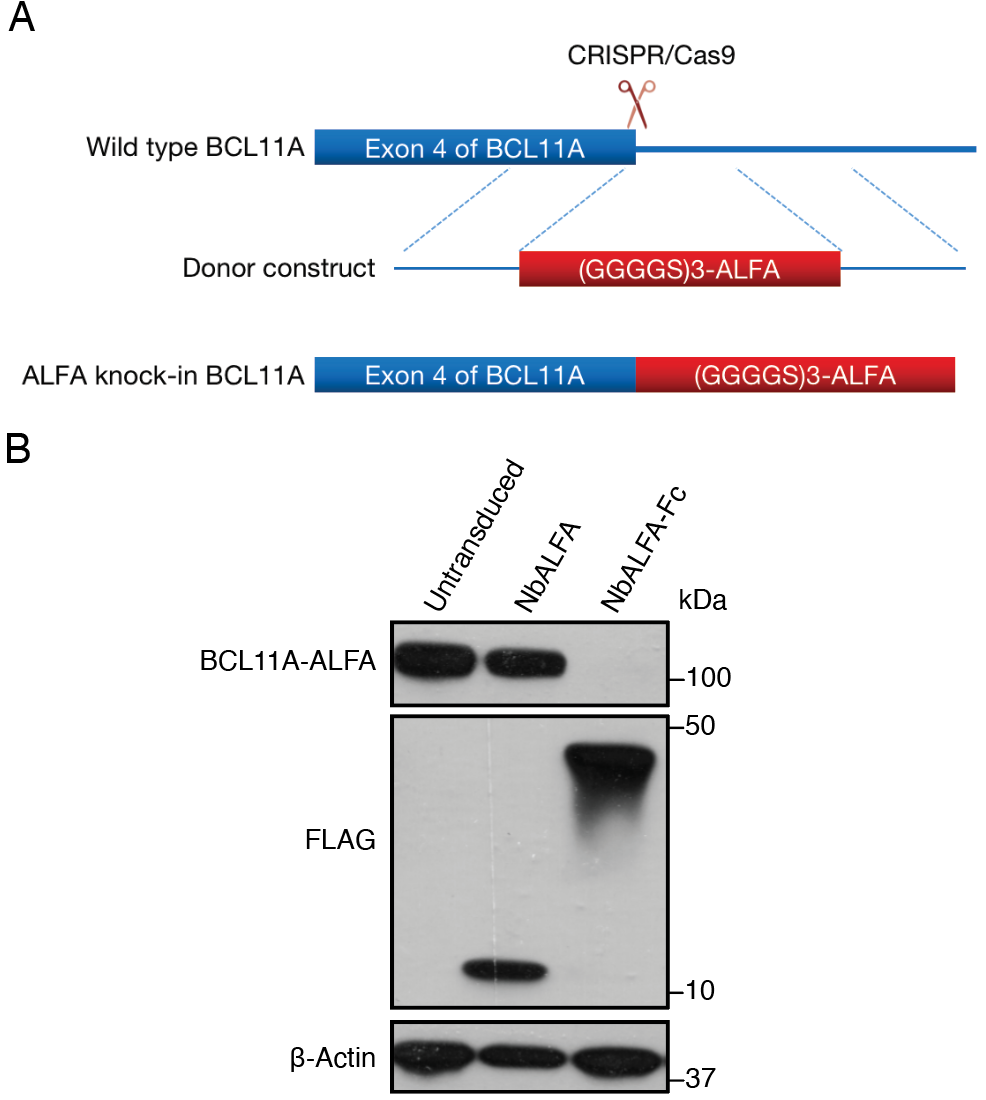


Figure S4. Generation of biallelic BCL11A-ALFA knock-in HUDEP2 cells. A, BCL11A-ALFA knock-in strategy. The stop codon of BCL11A was replaced with an in-frame (GGGGS)3-ALFA cassette. B, Validation of BCL11A-ALFA knock-in HUDEP2 cells by Trim-Away (37). Compared to untransduced cells, NbALFA-Fc fusion protein degraded BCL11A-ALFA fusion protein, but not the NbALFA protein only. NbALFA-Fc and NbALFA proteins were tagged with FLAG to detect their expression levels.

Supplementary Figure S5


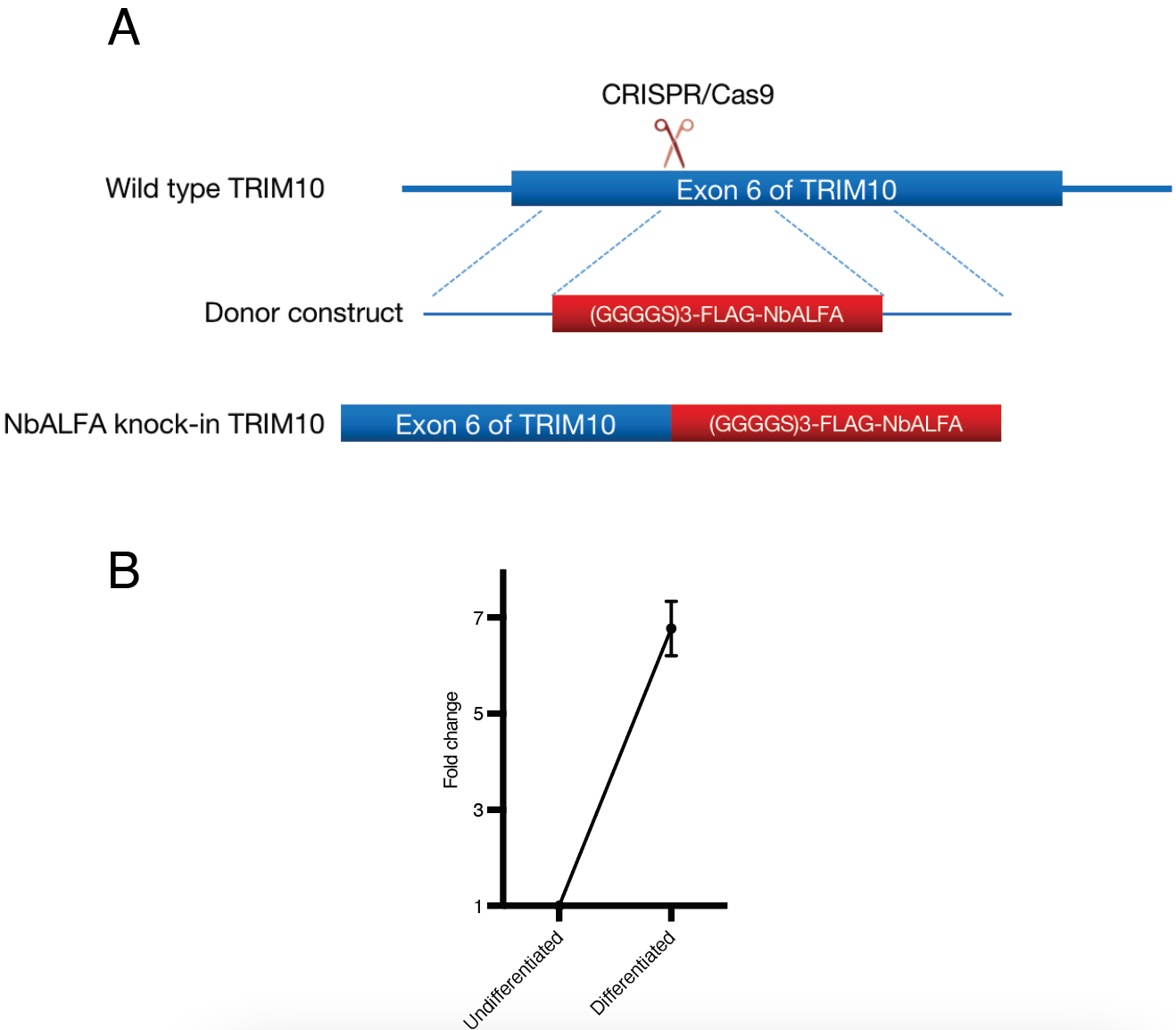


Figure S5. Generation of monoallelic TRIM10-NbALFA knock-in cells based on BCL11A-ALFA HUDEP2 cells. A, TRIM10-NbALFA knock-in strategy. FLAG-NbALFA cassette was inserted before the PRY/SPRY domain. B, the fold change of TRIM10 RNA level during the differentiation of HUDEP2 cells.

Supplementary Table S1

Table S1. Cell culture media

| Medium | Ingredients |
| --- | --- |
| CD34 expansion medium | Stemspan SFEM, 100 U/mL Penicillin, 100 ug/mL Streptomycin, 2 mM L-glutamine, 100 ng/mM rhFlt3-Ligand, 100 ng/mL rhSCF, 50 ng/mL rhTPO, 20 ng/mL rhIL3, 20 ng/mL rhIL6, 100nM Dexamethasone |
| CD34 differentiation medium 1 | IMDM, 100 U/mL Penicillin, 100 ug/mL Streptomycin, 2 mM L-glutamine, 5% human AB blood plasma, 3 U/ml Heparin, 10 μg/ml Insulin, 200 μg/ml holo-transferrin, 10 ng/ml rhSCF, 1 ng/ml rhIL3, 3 U/ml Epo |
| CD34 differentiation medium 2 | IMDM, 100 U/mL Penicillin, 100 ug/mL Streptomycin, 2 mM L-glutamine, 5% human AB blood plasma, 3 U/ml Heparin, 10 μg/ml Insulin, 200 μg/ml holo-transferrin, 10 ng/ml rhSCF, 1 U/ml Epo |
| HEK293T medium | DMEM supplemented with 10% FCS, 100 U/mL Penicillin, 100 ug/mL Streptomycin and 2 mM L-glutamine |
| HUDEP2 expansion medium | Stemspan SFEM, 200 U/mL Penicillin, 200 µg/mL Streptomycin, 100 ng/mL rhSCF, 3 IU/mL Epo, 10µM Dexamethasone, 1 µg/mL doxycycline |
| HUDEP2 differentiation medium | IMDM, 200 U/mL Penicillin, 200 ug/mL Streptomycin, 2 mM L-glutamine, 330 μg/mL human holo-transferrin, 10 μg/mL recombinant human insulin solution, 2 IU/mL heparin, 5% inactivated human plasma, 3 IU/mL Epo, 100 ng/mL rhSCF, 1 µg/mL doxycycline |

Supplementary Table S2

Table S2. qPCR primers

| TRIM10-forward | gtgagggtgtctcttgactatg |
| --- | --- |
| TRIM10-reverse | agagcccaaagaagggaatg |
| TRIM58-forward | gaaagagttggaggacgccttgactc |
| TRIM58-reverse | tcccgcagtctctgcagcgtcgctc |
| CRBN-forward | caccctcaagaagtcagtatgg |
| CRBN-reverse | caaactgtgcttccctttcctgtac |
| VHL-forward | atgttgacggacagcctatttttg |
| VHL-reverse | cgatgtccagtctcctgtaattctc |
| GAPDH-forward | acccagaagactgtggatgg |
| GAPDH-reverse | ttcagctcagggatgacctt |
| HBB-forward | ctgaggagaagtctgccgtta |
| HBB-reverse | agcatcaggagtggacagat |
| HBG-forward | tggatgatctcaagggcac |
| HBG-reverse | tcagtggtatctggaggaca |

Supplementary Table S3

Table S3. Constructs of fusion proteins

| Construct | N-terminus tag | Gene | C-terminus recruiter |
| --- | --- | --- | --- |
| TRIM10/58-P1 | 3xHA | TRIM10 (1-303aa)/  TRIM58 (1-284aa) | Coiled-coil peptide P1 |
| BCL11A-P2 | 3xFLAG | BCL11A | Coiled-coil peptide P2 |
| TRIM10/58-NbALFA | 3xHA | TRIM10 (1-303aa)/  TRIM58 (1-284aa) | NbALFA |
| BCL11A-ALFA | 3xFLAG | BCL11A | ALFA tag |
| TRIM10/58-Nb19 | 3xHA | TRIM10 (1-303aa)/  TRIM58 (1-284aa) | Nb19 |
| BCL11A | 3xFALG | BCL11A | N/A |
| TRIM10/58-CULT | 3xHA | TRIM10 (1-303aa)/  TRIM58 (1-284aa) | CRBN CULT |
| BCL11A-FKBP12^F36V^ | 3xFLAG | BCL11A | BCL11A-FKBP12^F36V^ |

Supplementary Table S4

Table S4. Antibodies

| Antibody | Vendor, catalog number |
| --- | --- |
| anti-HA | Abcam, ab236632 |
| anti-BCL11A | Abcam, ab191401 |
| anti-MTA2 | Abcam, ab8106 |
| anti-CHD4 | Abcam, ab72418 |
| anti-GATA1 | Abcam, ab11852 |
| anti-FLAG-HRP | Sigma-Aldrich, A8592 |
| anti-GAPDH-HRP | Invitrogen, MA5-44678 |
| anti-β-Actin-HRP | Santa Cruz, sc-47778 HRP |
| anti-Rabbit IgG-HRP | Invitrogen, 31460 |
| anti-Rabbit IgG-FITC | Thermo Fiserm, 65-6111 |

Supplementary Table S5

Table S5. gRNAs for electroporation

| Purpose | Gene | gRNA sequence | Nucleofector Solution and Supplement | Program |
| --- | --- | --- | --- | --- |
| knock-out | CRBN | GTCCTGCTGATCTCCTTCGC | SF Cell Line 4D-Nucleofector™ X Kit  (Lonza) | CM-130 |
| knock-in | BCL11A | ATGATATAAAAACTGAATAG | P3 Primary Cell 4D-Nucleofector^TM^ X Kit (Lonza) | EO-100 |
| knock-in | TRIM10 | GTTGGACTATGAGCCAGGTA |  |  |
| knock-out | TRIM10 | ATCTGCCAGGCTGGTCACAG |  |  |

Supplementary Table S6

Table S6. Primers for genotyping of genomic modification

| Primer | Sequence | Size of PCR product | |
| --- | --- | --- | --- |
|  |  | Wild type allele | Modified allele |
| CRBN-genotyping-forward | ccggtgcgcggcagccgcgcgacac | 273bp | 415bp |
| CRBN-genotyping-reverse | ctgctgggctggctcgccaggcttg |  |  |
| BCL11A-ALFA-genotyping-forward | gtgaaatttgtaagatgccttttagcg | 280bp | 367bp |
| BCL11A-ALFA-genotyping-reverse | ggtgacaagcactctcatattcttagc |  |  |
| TRIM10-NbALFA-genotyping-forward | ctttctgttagtctggctcagagttagac | 1564bp | 2039bp |
| TRIM10-NbALFA-genotyping-reverse | cccagggcactggtgactcatttacag |  |  |
